# *N*-(2-Meth­oxy­benz­yl)-9-(oxolan-2-yl)-9*H*-purin-6-amine

**DOI:** 10.1107/S1600536813007721

**Published:** 2013-03-23

**Authors:** Zdeněk Trávníček, Igor Popa, Zdeněk Dvořák, Pavel Štarha

**Affiliations:** a Department of Inorganic Chemistry, Faculty of Science, Palacký University, 17. listopadu 12, CZ-771 46 Olomouc, Czech Republic; b Department of Cell Biology and Genetics, Faculty of Science, Palacký University, Šlechtitelů 11, CZ-783 71 Olomouc, Czech Republic

## Abstract

The title compound, C_17_H_19_N_5_O_2_, features an almost planar purine skeleton (r.m.s. deviation = 0.009 Å) substituted by a tetra­hydro­furan ring, which adopts an envelope conformation. The purine and benzene rings subtend a dihedral angle of 66.70 (3)°. In the crystal, pairs of N—H⋯N hydrogen bonds connect adjacent mol­ecules into inversion dimers. C—H⋯N, C—H⋯O, C—H⋯π and π–π inter­actions [pyrimidine ring centroid–centroid distance = 3.3909 (1) Å] connect the dimers into a three-dimensional architecture.

## Related literature

For an alternative synthetic procedure and the biological activity of benzyl-substituted 6-benzyl­amino-9-tetra­hydro­pyran-2-yl-9*H*-purine derivatives, see: Szüčová *et al.* (2009 ▶). For a related structure, see: Štarha *et al.* (2013 ▶).
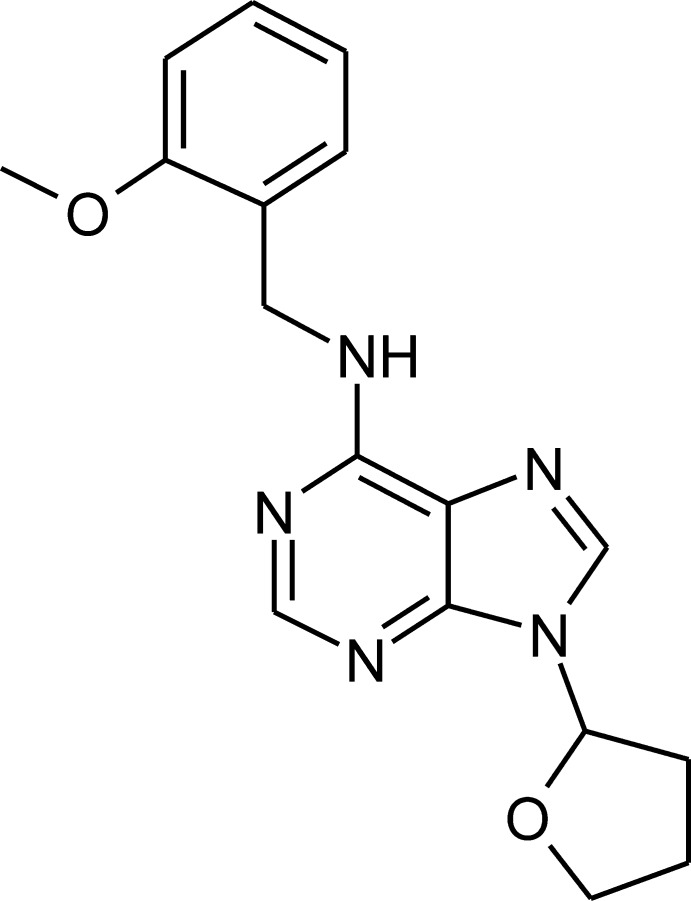


## Experimental

### 

#### Crystal data

C_17_H_19_N_5_O_2_
*M* *_r_* = 325.37
Monoclinic, 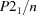

*a* = 8.87210 (19) Å
*b* = 8.37534 (17) Å
*c* = 20.7445 (4) Å
β = 90.4360 (19)°
*V* = 1541.42 (6) Å^3^
*Z* = 4
Mo *K*α radiation
μ = 0.10 mm^−1^
*T* = 110 K
0.35 × 0.30 × 0.30 mm

#### Data collection

Agilent Xcalibur Sapphire2 diffractometer
Absorption correction: multi-scan (*CrysAlis PRO*; Agilent, 2012 ▶) *T*_min_ = 0.967, *T*_max_ = 0.972
12687 measured reflections
2719 independent reflections
2415 reflections with *I* > 2σ(*I*)
*R*_int_ = 0.014

#### Refinement

*R*[*F* ^2^ > 2σ(*F* ^2^)] = 0.035
*wR*(*F* ^2^) = 0.087
*S* = 1.04
2719 reflections
218 parameters
H-atom parameters constrained
Δρ_max_ = 0.37 e Å^−3^
Δρ_min_ = −0.23 e Å^−3^

### 

Data collection: *CrysAlis PRO* (Agilent, 2012 ▶); cell refinement: *CrysAlis PRO*; data reduction: *CrysAlis PRO*; program(s) used to solve structure: *SHELXS97* (Sheldrick, 2008 ▶); program(s) used to refine structure: *SHELXL97* (Sheldrick, 2008 ▶); molecular graphics: *DIAMOND* (Brandenburg, 2011 ▶); software used to prepare material for publication: *publCIF* (Westrip, 2010 ▶).

## Supplementary Material

Crystal structure: contains datablock(s) I, global. DOI: 10.1107/S1600536813007721/ng5319sup1.cif

Structure factors: contains datablock(s) I. DOI: 10.1107/S1600536813007721/ng5319Isup2.hkl

Supplementary material file. DOI: 10.1107/S1600536813007721/ng5319Isup3.cml

Additional supplementary materials:  crystallographic information; 3D view; checkCIF report

## Figures and Tables

**Table 1:** Hydrogen-bond geometry (Å, °) *Cg* is the centroid of the C10–C15 ring.

*D*—H⋯*A*	*D*—H	H⋯*A*	*D*⋯*A*	*D*—H⋯*A*
N6—H6⋯N7^i^	0.88	2.32	3.145 (2)	157
C8—H8⋯*Cg* ^i^	0.95	2.86	3.6214 (14)	138
C12—H12⋯O2^ii^	0.95	2.60	3.459 (2)	150
C13—H13⋯N3^ii^	0.95	2.55	3.489 (2)	170

Symmetry codes: (i) 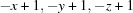; (ii) 
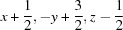
.

**Fig. 1.**
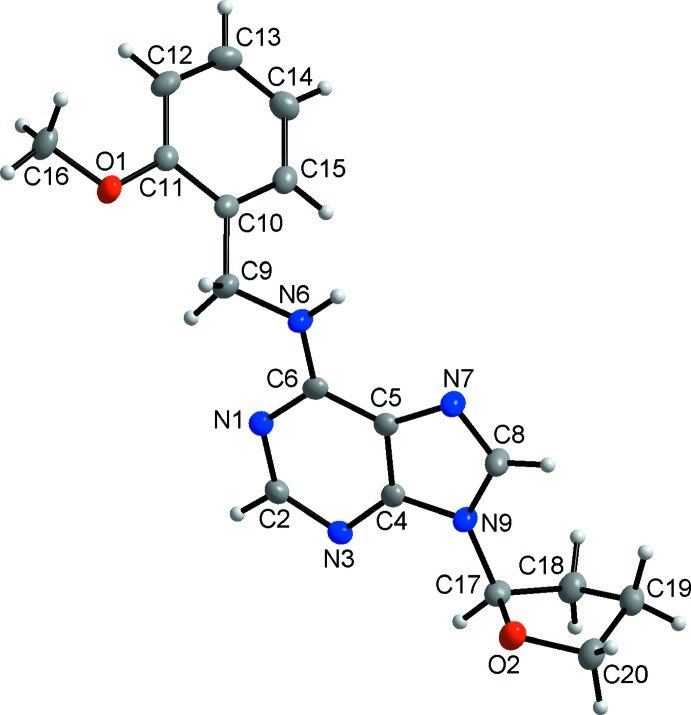
The molecular structure of the title compound with the non-hydrogen atoms depicted as thermal ellipsoids at the 50% probability level and given with the atom numbering scheme.

**Fig. 2.**
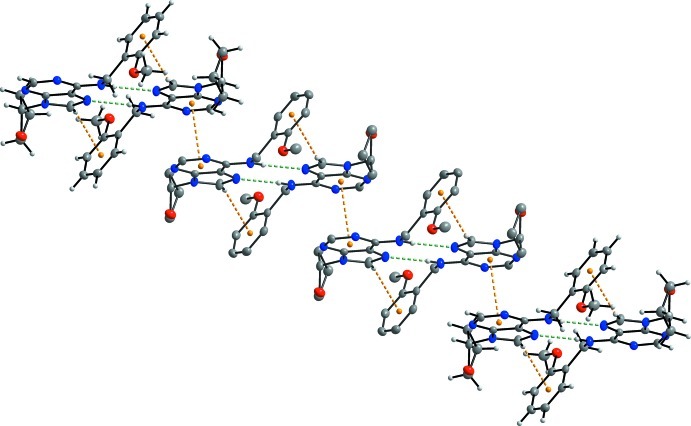
Part of the crystal structure, showing the N6—H6···N7 hydrogen bonds (dashed green lines; see Table 1 for parameters), C8—H8···π interactions (dashed orange lines; see Table 1 for C8—H8···Cg parameters) and π—π interactions (dashed orange lines; the Cg—Cgi distance = 3.39090 (10) Å).

## supplementary crystallographic information

### Comment

The molecule of *N*-(2-methoxybenzyl)-9-(oxolan-2-yl)-9*H*-purin-6-amine consists of six-membered pyrimidine and five-membered imidazole rings merged to the essentially planar purine skeleton, which is substituted by 2-methoxybenzylamine and oxolan-2-yl at the C6, and N9 position, respectively (Figure 1). Two N6—H6···N7 hydrogen bonds (Table 1) connect the molecules into the centrosymmetric dimers (Figure 2) with coplanar purine moieties (dihedral angle of 0.00 (3)°). Except for the hydrogen bonds, the C—H···N, C—H···O, C—H···π and π–π interactions (Figure 2) also link the individual molecules within the crystal structure into a three-dimensional architecture. The planar pyrimidine (the most deviated atoms from the LSQ-plane fitted through its atoms: C5, 0.0122 (13) Å) and imidazole (the most deviated atom from the LSQ-plane fitted through its atoms: C8, -0.002 (2) Å) rings of the purine moiety form the dihedral angle of 0.72 (4)°. The planes fitted through the atoms of the purine and benzene rings form the dihedral angle of 66.70 (3)°.

### Experimental

*N*-(2-methoxybenzyl)-9-(oxolan-2-yl)-9*H*-purin-6-amine, a perspective ligand of the transition metal complexes, was synthesized by a modification of the recently reported method (Szüčová *et al.*, 2009). 6-Chloropurine reacted with 2,3-dihydrofurane in a molar ratio of 1:2 for 15 min at laboratory temperature in a minimum volume of ethanol, followed by the addition of CF_3_COOH (1.30 molar equivalent of 6-chloropurine). The mixture was stirred at laboratory temperature for 24 h and after that it was neutralized by 10% NH_4_OH, evaporated to dryness, washed by distilled water, methanol and diethyl ether and dried in desiccator over P_4_O_10_. The obtained intermediate, *i.e.* 6-chloro-9-(oxolan-2-yl)-9*H*-purine interacted with 2-methoxybenzylamine and triethylamine (molar ratio of 1: 1.33: 1.67, respectively) in *N,N`*-dimethylformamide (90 °C, 150 min). Again, the solvents were partly evaporated and the obtained product was separated by filtration after it was suspended in distilled water. The title compound was washed with distilled water, methanol and diethyl ether and dried (in a desiccator over P_4_O_10_). Single-crystals were prepared by recrystallization of the product from ethanol. Analysis calculated for C_17_H_19_N_5_O_2_: C 62.8, H 5.9, N 21.5%; found: C 62.6, H 6.1, N 21.2%. Elemental analysis (C, H, N) was performed on a Thermo Scientific Flash 2000 CHNO-S Analyzer.

### Refinement

Non-hydrogen atoms were refined anisotropically and hydrogen atoms were located in difference maps and refined using the riding model with C—H = 0.95 (CH), C—H = 0.99 (CH_2_), C—H = 0.98 (CH_3_) Å, and N—H = 0.88 Å, with *U*_iso_(H) = 1.2*U*_eq_(CH, CH_2_, NH) and 1.5*U*_eq_(CH_3_). The maximum and minimum residual electron density peaks of 0.37 and -0.23 e Å^-3^ were located 0.81 Å, and 0.72 Å from the H18A, and C17 atoms, respectively.

### Crystal data

**Table:**

C_17_H_19_N_5_O_2_	*F*(000) = 688
*M**_r_* = 325.37	*D*_x_ = 1.402 Mg m^−^^3^
Monoclinic, *P*2_1_/*n*	Mo *K*α radiation, λ = 0.71073 Å
Hall symbol: -P 2yn	Cell parameters from 11022 reflections
*a* = 8.87210 (19) Å	θ = 2.9–31.9°
*b* = 8.37534 (17) Å	µ = 0.10 mm^−^^1^
*c* = 20.7445 (4) Å	*T* = 110 K
β = 90.4360 (19)°	Prism, colourless
*V* = 1541.42 (6) Å^3^	0.35 × 0.30 × 0.30 mm
*Z* = 4	

### Data collection

**Table:**

Agilent Xcalibur Sapphire2 diffractometer	2719 independent reflections
Radiation source: Enhance (Mo) X-ray Source	2415 reflections with *I* > 2σ(*I*)
Graphite monochromator	*R*_int_ = 0.014
Detector resolution: 8.3611 pixels mm^-1^	θ_max_ = 25.0°, θ_min_ = 3.1°
ω scans	*h* = −9→10
Absorption correction: multi-scan (*CrysAlis PRO*; Agilent, 2012)	*k* = −9→9
*T*_min_ = 0.967, *T*_max_ = 0.972	*l* = −23→24
12687 measured reflections	

### Refinement

**Table:**

Refinement on *F*^2^	Primary atom site location: structure-invariant direct methods
Least-squares matrix: full	Secondary atom site location: difference Fourier map
*R*[*F*^2^ > 2σ(*F*^2^)] = 0.035	Hydrogen site location: inferred from neighbouring sites
*wR*(*F*^2^) = 0.087	H-atom parameters constrained
*S* = 1.04	*w* = 1/[σ^2^(*F*_o_^2^) + (0.0396*P*)^2^ + 0.7716*P*] where *P* = (*F*_o_^2^ + 2*F*_c_^2^)/3
2719 reflections	(Δ/σ)_max_ = 0.001
218 parameters	Δρ_max_ = 0.37 e Å^−^^3^
0 restraints	Δρ_min_ = −0.23 e Å^−^^3^

### Special details

**Table:**

Geometry. All e.s.d.'s (except the e.s.d. in the dihedral angle between two l.s. planes) are estimated using the full covariance matrix. The cell e.s.d.'s are taken into account individually in the estimation of e.s.d.'s in distances, angles and torsion angles; correlations between e.s.d.'s in cell parameters are only used when they are defined by crystal symmetry. An approximate (isotropic) treatment of cell e.s.d.'s is used for estimating e.s.d.'s involving l.s. planes.
Refinement. Refinement of *F*^2^ against ALL reflections. The weighted *R*-factor *wR* and goodness of fit *S* are based on *F*^2^, conventional *R*-factors *R* are based on *F*, with *F* set to zero for negative *F*^2^. The threshold expression of *F*^2^ > σ(*F*^2^) is used only for calculating *R*-factors(gt) *etc*. and is not relevant to the choice of reflections for refinement. *R*-factors based on *F*^2^ are statistically about twice as large as those based on *F*, and *R*- factors based on ALL data will be even larger.

### Fractional atomic coordinates and isotropic or equivalent isotropic displacement parameters (Å^2^)

**Table:**

	*x*	*y*	*z*	*U*_iso_*/*U*_eq_	
N1	0.08392 (13)	0.72477 (14)	0.53161 (5)	0.0216 (3)	
O1	0.39104 (11)	1.15624 (12)	0.40650 (5)	0.0265 (2)	
O2	0.14507 (12)	0.19144 (12)	0.72047 (5)	0.0295 (3)	
C2	−0.02259 (15)	0.65607 (17)	0.56768 (6)	0.0214 (3)	
H2	−0.1142	0.7142	0.5709	0.026*	
N3	−0.01895 (12)	0.51849 (14)	0.59968 (5)	0.0208 (3)	
C4	0.11411 (15)	0.44422 (16)	0.59082 (6)	0.0184 (3)	
C5	0.23360 (15)	0.49756 (16)	0.55428 (6)	0.0188 (3)	
N6	0.32579 (13)	0.71553 (14)	0.48978 (6)	0.0241 (3)	
H6	0.4078	0.6597	0.4822	0.029*	
C6	0.21608 (15)	0.64750 (16)	0.52444 (6)	0.0190 (3)	
N7	0.35162 (13)	0.38842 (14)	0.55594 (6)	0.0241 (3)	
C8	0.30091 (16)	0.27427 (18)	0.59319 (7)	0.0257 (3)	
H8	0.3582	0.1818	0.6036	0.031*	
N9	0.15811 (12)	0.29976 (14)	0.61589 (6)	0.0216 (3)	
C9	0.31619 (17)	0.87629 (17)	0.46427 (7)	0.0247 (3)	
H9A	0.3913	0.9444	0.4867	0.030*	
H9B	0.2149	0.9201	0.4733	0.030*	
C10	0.34376 (14)	0.88340 (16)	0.39253 (7)	0.0197 (3)	
C11	0.38461 (14)	1.02956 (17)	0.36481 (7)	0.0203 (3)	
C12	0.41548 (16)	1.03960 (19)	0.29956 (7)	0.0257 (3)	
H12	0.4460	1.1383	0.2812	0.031*	
C13	0.40157 (16)	0.90491 (19)	0.26119 (7)	0.0291 (4)	
H13	0.4219	0.9119	0.2164	0.035*	
C14	0.35861 (16)	0.76098 (19)	0.28742 (7)	0.0270 (3)	
H14	0.3484	0.6692	0.2608	0.032*	
C15	0.33028 (15)	0.75093 (17)	0.35321 (7)	0.0228 (3)	
H15	0.3013	0.6515	0.3713	0.027*	
C16	0.42614 (18)	1.30910 (18)	0.38005 (8)	0.0307 (4)	
H16A	0.4216	1.3901	0.4141	0.046*	
H16B	0.3531	1.3354	0.3460	0.046*	
H16C	0.5278	1.3067	0.3619	0.046*	
C17	0.06967 (16)	0.20020 (17)	0.66006 (7)	0.0242 (3)	
H17	−0.0313	0.2506	0.6663	0.029*	
C18	0.04761 (17)	0.02969 (18)	0.63684 (7)	0.0278 (3)	
H18A	0.0622	0.0218	0.5897	0.033*	
H18B	−0.0544	−0.0098	0.6475	0.033*	
C19	0.16852 (17)	−0.06373 (18)	0.67324 (7)	0.0275 (3)	
H19A	0.1373	−0.1757	0.6805	0.033*	
H19B	0.2656	−0.0625	0.6500	0.033*	
C20	0.17883 (17)	0.02796 (18)	0.73579 (7)	0.0292 (4)	
H20A	0.1055	−0.0142	0.7672	0.035*	
H20B	0.2814	0.0190	0.7546	0.035*	

### Atomic displacement parameters (Å^2^)

**Table:**

	*U*^11^	*U*^22^	*U*^33^	*U*^12^	*U*^13^	*U*^23^
N1	0.0222 (6)	0.0213 (6)	0.0211 (6)	0.0008 (5)	0.0011 (5)	0.0005 (5)
O1	0.0342 (6)	0.0177 (5)	0.0276 (5)	−0.0020 (4)	0.0020 (4)	0.0026 (4)
O2	0.0407 (6)	0.0234 (6)	0.0245 (5)	0.0000 (5)	0.0005 (5)	0.0013 (4)
C2	0.0208 (7)	0.0209 (7)	0.0224 (7)	0.0018 (6)	0.0021 (5)	−0.0016 (6)
N3	0.0202 (6)	0.0216 (6)	0.0207 (6)	0.0003 (5)	0.0022 (5)	−0.0009 (5)
C4	0.0198 (7)	0.0182 (7)	0.0171 (6)	−0.0017 (5)	−0.0017 (5)	−0.0016 (5)
C5	0.0191 (7)	0.0202 (7)	0.0170 (7)	−0.0011 (5)	0.0000 (5)	−0.0010 (5)
N6	0.0225 (6)	0.0234 (6)	0.0265 (6)	0.0029 (5)	0.0064 (5)	0.0077 (5)
C6	0.0220 (7)	0.0208 (7)	0.0142 (6)	−0.0023 (6)	−0.0002 (5)	−0.0015 (5)
N7	0.0204 (6)	0.0221 (6)	0.0298 (7)	0.0009 (5)	0.0041 (5)	0.0043 (5)
C8	0.0191 (7)	0.0223 (8)	0.0360 (8)	0.0020 (6)	0.0042 (6)	0.0061 (6)
N9	0.0181 (6)	0.0202 (6)	0.0266 (6)	−0.0002 (5)	0.0023 (5)	0.0046 (5)
C9	0.0286 (8)	0.0219 (8)	0.0237 (7)	−0.0017 (6)	0.0036 (6)	0.0044 (6)
C10	0.0137 (6)	0.0223 (7)	0.0232 (7)	0.0020 (5)	0.0012 (5)	0.0039 (6)
C11	0.0153 (6)	0.0210 (7)	0.0246 (7)	0.0004 (5)	−0.0011 (5)	0.0018 (6)
C12	0.0228 (7)	0.0290 (8)	0.0254 (7)	−0.0026 (6)	0.0003 (6)	0.0092 (6)
C13	0.0263 (8)	0.0407 (9)	0.0202 (7)	−0.0004 (7)	0.0012 (6)	0.0022 (7)
C14	0.0234 (7)	0.0309 (8)	0.0267 (8)	0.0001 (6)	−0.0017 (6)	−0.0049 (6)
C15	0.0166 (7)	0.0213 (7)	0.0303 (8)	0.0003 (5)	−0.0008 (6)	0.0033 (6)
C16	0.0365 (9)	0.0194 (8)	0.0363 (9)	−0.0038 (6)	−0.0038 (7)	0.0071 (6)
C17	0.0186 (7)	0.0244 (8)	0.0296 (8)	−0.0002 (6)	0.0026 (6)	0.0041 (6)
C18	0.0262 (8)	0.0239 (8)	0.0334 (8)	−0.0044 (6)	−0.0024 (6)	0.0023 (7)
C19	0.0269 (8)	0.0207 (8)	0.0350 (8)	0.0009 (6)	0.0006 (6)	0.0015 (6)
C20	0.0298 (8)	0.0242 (8)	0.0334 (8)	0.0004 (6)	−0.0019 (6)	0.0043 (7)

### Geometric parameters (Å, º)

**Table:**

N1—C2	1.3396 (18)	C10—C15	1.382 (2)
N1—C6	1.3485 (18)	C10—C11	1.4014 (19)
O1—C11	1.3698 (17)	C11—C12	1.385 (2)
O1—C16	1.4282 (17)	C12—C13	1.386 (2)
O2—C17	1.4179 (18)	C12—H12	0.9500
O2—C20	1.4366 (18)	C13—C14	1.377 (2)
C2—N3	1.3302 (18)	C13—H13	0.9500
C2—H2	0.9500	C14—C15	1.392 (2)
N3—C4	1.3482 (17)	C14—H14	0.9500
C4—N9	1.3725 (18)	C15—H15	0.9500
C4—C5	1.3821 (19)	C16—H16A	0.9800
C5—N7	1.3902 (17)	C16—H16B	0.9800
C5—C6	1.4082 (19)	C16—H16C	0.9800
N6—C6	1.3414 (17)	C17—C18	1.519 (2)
N6—C9	1.4490 (18)	C17—H17	1.0000
N6—H6	0.8800	C18—C19	1.523 (2)
N7—C8	1.3111 (19)	C18—H18A	0.9900
C8—N9	1.3716 (18)	C18—H18B	0.9900
C8—H8	0.9500	C19—C20	1.510 (2)
N9—C17	1.4701 (18)	C19—H19A	0.9900
C9—C10	1.5111 (19)	C19—H19B	0.9900
C9—H9A	0.9900	C20—H20A	0.9900
C9—H9B	0.9900	C20—H20B	0.9900
			
C2—N1—C6	118.24 (12)	C11—C12—H12	120.2
C11—O1—C16	117.38 (11)	C14—C13—C12	120.62 (13)
C17—O2—C20	109.94 (11)	C14—C13—H13	119.7
N3—C2—N1	129.52 (13)	C12—C13—H13	119.7
N3—C2—H2	115.2	C13—C14—C15	119.51 (14)
N1—C2—H2	115.2	C13—C14—H14	120.2
C2—N3—C4	110.46 (11)	C15—C14—H14	120.2
N3—C4—N9	127.00 (12)	C10—C15—C14	120.98 (13)
N3—C4—C5	126.99 (13)	C10—C15—H15	119.5
N9—C4—C5	106.00 (12)	C14—C15—H15	119.5
C4—C5—N7	110.77 (12)	O1—C16—H16A	109.5
C4—C5—C6	116.54 (12)	O1—C16—H16B	109.5
N7—C5—C6	132.67 (12)	H16A—C16—H16B	109.5
C6—N6—C9	123.33 (12)	O1—C16—H16C	109.5
C6—N6—H6	118.3	H16A—C16—H16C	109.5
C9—N6—H6	118.3	H16B—C16—H16C	109.5
N6—C6—N1	119.37 (12)	O2—C17—N9	109.25 (11)
N6—C6—C5	122.43 (12)	O2—C17—C18	106.88 (11)
N1—C6—C5	118.20 (12)	N9—C17—C18	113.81 (12)
C8—N7—C5	103.43 (11)	O2—C17—H17	108.9
N7—C8—N9	114.26 (13)	N9—C17—H17	108.9
N7—C8—H8	122.9	C18—C17—H17	108.9
N9—C8—H8	122.9	C17—C18—C19	103.72 (12)
C8—N9—C4	105.54 (11)	C17—C18—H18A	111.0
C8—N9—C17	128.64 (12)	C19—C18—H18A	111.0
C4—N9—C17	125.77 (11)	C17—C18—H18B	111.0
N6—C9—C10	112.76 (12)	C19—C18—H18B	111.0
N6—C9—H9A	109.0	H18A—C18—H18B	109.0
C10—C9—H9A	109.0	C20—C19—C18	101.68 (12)
N6—C9—H9B	109.0	C20—C19—H19A	111.4
C10—C9—H9B	109.0	C18—C19—H19A	111.4
H9A—C9—H9B	107.8	C20—C19—H19B	111.4
C15—C10—C11	118.73 (12)	C18—C19—H19B	111.4
C15—C10—C9	122.39 (12)	H19A—C19—H19B	109.3
C11—C10—C9	118.88 (12)	O2—C20—C19	106.47 (12)
O1—C11—C12	124.21 (13)	O2—C20—H20A	110.4
O1—C11—C10	115.28 (12)	C19—C20—H20A	110.4
C12—C11—C10	120.51 (13)	O2—C20—H20B	110.4
C13—C12—C11	119.63 (13)	C19—C20—H20B	110.4
C13—C12—H12	120.2	H20A—C20—H20B	108.6

### Hydrogen-bond geometry (Å, º)

Cg is the centroid of the C10–C15 ring.

**Table:**

*D*—H···*A*	*D*—H	H···*A*	*D*···*A*	*D*—H···*A*
N6—H6···N7^i^	0.88	2.32	3.145 (2)	157
C8—H8···*Cg*^i^	0.95	2.86	3.6214 (14)	138
C12—H12···O2^ii^	0.95	2.60	3.459 (2)	150
C13—H13···N3^ii^	0.95	2.55	3.489 (2)	170

Symmetry codes: (i) −*x*+1, −*y*+1, −*z*+1; (ii) *x*+1/2, −*y*+3/2, *z*−1/2.
